# Degradation Mechanism and Numerical Simulation of Pervious Concrete under Salt Freezing-Thawing Cycle

**DOI:** 10.3390/ma15093054

**Published:** 2022-04-22

**Authors:** Junzheng Xiang, Hengrui Liu, Hao Lu, Faliang Gui

**Affiliations:** 1 College of Water Conservancy and Hydropower Engineering, Hohai University, Xikang Road No. 1, Nanjing 210098, China; junzhengx@163.com (J.X.); 180402020005@hhu.edu.cn (H.L.); 2 School of Hydraulic & Ecological Engineering, Nanchang Institute of Technology, Nanchang 330099, China; guifaliang@126.com

**Keywords:** pervious concrete, freeze-thaw cycle, deterioration law, interfacial transition zone (ITZ) of hardened paste and aggregate, discrete element simulation

## Abstract

In order to explore the occurrence area of pervious concrete freeze-thaw deterioration, the mass loss, strength deterioration, ultrasonic longitudinal wave velocity and dynamic elastic modulus attenuation of pervious concrete under freeze-thaw cycles were measured, and a prediction model of freeze-thaw damage was established. The mechanical properties of hardened cement pastes with the same W/C ratio under freeze-thaw cycles were also measured. Mercury intrusion porosimetry (MIP) was used to measure the pore structure characteristic parameters and pore size distribution changes of cement paste under freeze-thaw cycle, and the microstructure evolution of interfacial transition zone (ITZ) of paste and aggregate was observed by SEM scanning electron microscopy. Finally, a pervious concrete model was established by DEM to analyze the relationship between the number of freeze-thaw cycles and the mesoscopic parameters. The results indicated that the quality, strength and dynamic elastic modulus of pervious concrete deteriorate to different degrees under the conditions of water freezing and salt freezing. The damage sensitivity and strength loss of freeze-thaw damage is greater than the dynamic elastic modulus loss, which is greater than mass loss. In the pervious concrete paste which underwent 100 freeze-thaw cycles, the pore structure and macro strength had no obvious change, and hardened paste and the aggregate-interface-generated defects increased with the increase in freezing and thawing times, indicating that the deterioration of pervious concrete performance under freeze-thaw cycles was closely related to the deterioration of the interface strength of the aggregate and hardened paste. The pervious concrete model established by DEM can accurately simulate the change of the compressive modulus and the strength of pervious concrete during freeze-thaw cycles.

## 1. Introduction

Pervious concrete is a porous and lightweight concrete made of aggregate, cement, reinforcer, mineral admixture and water. As the surface of the coarse aggregate is covered with thin layers of cement paste and it bonds to itself, a honeycomb structure is formed with evenly distributed pores; this gives pervious concrete the characteristics of air permeability, water permeability and light weight, and is widely used in the engineering field [1,2,3]. However, pervious concrete also has the characteristics of easy clogging and low strength, which is damaged more by freezing and thawing in a cold environment than ordinary concrete [4,5]. In cold conditions, pervious concrete also faces the double damage of salt freezing and chemical erosion; these factors affected its large-scale promotion and application in cold areas [6].

The frost resistance of pervious concrete increased with the decrease in aggregate particle size, increased with the increase in paste to aggregate mass ratio, and decreased significantly with the increase in water–cement ratio [7,8]. For the external environment, deicing salts negatively affected the strength and durability of conventional concrete pavements. The porosity of pervious concrete may increase the surface area vulnerable to a chemical attack [9,10,11]. Previous studies have shown that the porosity of pervious concrete was negatively correlated with strength [12,13]. Therefore, the effects of different types of salts on pervious concrete have been studied by some researchers, including freeze-thaw cycles, dry–wet cycles, or immersing in deicing salt solutions of different concentrations and temperatures. Concrete soaked in a sodium chloride solution had no significant negative effect on the dry–wet cycle, while calcium chloride and magnesium chloride (MgCl_2_) did significant damage to the mass loss and stiffness reduction of concrete [14]. The three deicing salts are sodium chloride, calcium chloride, and magnesium calcium acetate (CMA). Cutler et al. tested the freeze-thaw resistance of pervious concrete under saturated and unsaturated conditions. The damage degree was evaluated by measuring the mass loss and compressive strength periodically. It was found that calcium chloride caused the most damage, followed by sodium chloride and magnesium chloride, and the saturation test caused more damage than the unsaturated test [15]. Anderson et al. soaked pervious concrete samples in sodium chloride solutions of different concentration gradients and then discharged the solution for an unsaturated freeze-thaw cycle test. The results showed that 4% and 8% salt concentrations did the greatest damage to the denudation of cement paste, and did little damage to the aggregate [16]. Hassan Bilal et al. reported that the damage and mass loss caused by the combined actions of calcium leaching and freeze-thaw cycles were greater than those caused by freeze-thaw cycles and calcium leaching alone, and the combined erosion significantly increased the deterioration and damage of the pervious concrete [17].

Some researchers have improved the freeze-thaw resistance of pervious concrete under extreme cold conditions. Mehmet Gesoğlu et al. found that rubber was an effective solution to the freeze-thaw durability problem of pervious concrete. After 300 freeze-thaw cycles, rubber utilization significantly improved their results. The performance of small size rubber particles was better than that of large size rubber particles [18]. D. Tarangini et al. had proved that nano silica can increase the micro voids of pervious concrete, thus significantly increasing the freeze-thaw resistance of concrete, and this effect was better than other mineral admixtures [19]. Similarly, AoYang Li et al. reported that the addition of glass powder content resulted in a decrease in internal porosity and permeability coefficient of pervious concrete, which enhanced the freeze-thaw resistance [20]. In addition to mineral admixtures, other factors can also affect the freeze-thaw resistance of pervious concrete. Taheri B.M. et al. conducted tests and statistical analysis on the water–cement ratio (W/C), entrained gas volume, sand content, coarse aggregate particle size and other parameters that affected the freeze-thaw durability of pervious concrete, and believed that the strength and freeze-thaw durability of concrete could be improved by replacing 8% coarse aggregate with sand and a high W/C. It was found that the change of air entrainment and coarse aggregate particle size had no significant effect on the freeze-thaw durability of pervious concrete [21]. Rui Zhong et al. evaluated the influence of the matrix type, pore system characteristics and fiber on the F-T durability of the pervious concrete. The main influence of pore system characteristics on the F-T durability of PC was that of pore size and bending degree. Using a larger aggregate resulted in larger void sizes, higher bending, and premature F-T failure [22]. The correlation between the strength of pervious concrete and freeze-thaw durability was also explored. Pervious concrete with a higher strength usually had better durability, while tensile strength had a more significant impact on freeze-thaw durability than compressive strength. The tensile strength and freeze-thaw durability of pervious concrete were significantly enhanced by polypropylene fibers of different lengths (3~12 mm) [23]. Almeida et al. found that natural carbonization can improve the resistance of pervious concrete to deicing salts [24]. Bilal et al. added silica fume, metakaolin, and SBR (styrene-butadiene rubber) polymer emulsions to pervious concrete mixtures at different levels to improve their strength and durability. The results showed that the increase of supplementary cementitious materials (SCMs) from 5% to 10% significantly improved the resistance to rapid FT cycling [15]. Yang reported that the freeze-thaw durability of pervious concrete cured with saturated lime water was significantly higher than that of pervious concrete cured slowly in air. In addition, it was found that silica fume helped to improve the freeze-thaw resistance of pervious concrete cured with saturated lime water with deicing salts [25]. Most of the above studies were based on improving the properties of the cement base to improve the freeze-thaw durability of pervious concrete, and these methods also appeared in most studies of ordinary concrete [26,27,28,29,30,31].

However, compared with ordinary concrete, whether improving cement-based materials was the most effective way to improve frost resistance required further research on the freeze-thaw failure mechanism of pervious concrete. The mechanical behavior and failure mechanism of cement-based materials under freeze-thaw cycles has been studied by many scholars [32,33,34]. The ice formation began in the capillary pores of the cement matrix, and then unfrozen water was pushed around. The cracks appeared when the high expansion pressure exceeded the matrix strength and was not released in time [35]. When the water was impure, the water in the microscopic pores of the cementitious base contained high concentrations of salts used for deicing. After freezing, solutes may disrupt the chemical equilibrium in the connected pores, causing osmotic pressure disruption [36]. On the other hand, the water–cement ratio of pervious concrete was usually between 0.27 and 0.33, and the low water–cement ratio cement-based microstructure was more resistant to freezing and thawing. Vancura found cracks suspected of freezing and thawing from pervious concrete cores taken from a pavement in cold regions, and these cracks passed through the aggregate–cement interface [37]. Zhou et al. used two silane emulsion modification methods to change the properties of the recycled aggregate surface and cement matrix, thereby improving the freeze-thaw durability of recycled aggregate pervious concrete. The results showed that surface modification of the recycled aggregate can more effectively improve the compressive strength and freeze-thaw durability of recycled aggregate pervious concrete than overall modification of the cement matrix [35]. These results reflected that cement-based deterioration may not be the main cause of freeze-thaw deterioration of pervious concrete, but whether it was mainly from interface deterioration requires further research to analyze the microscopic mechanism of the permeable concrete’s freeze-thaw damage.

In recent years, many researchers have studied the freeze-thaw deterioration process of concrete through numerical simulations, such as the finite element method (FEM) and discrete element method (DEM), to deepen the understanding of this process [38,39,40,41]. The macro-mechanical behavior of general materials was determined by the meso-structural properties of the constituent materials, such as the strength and deformation properties of particles, the bonding strength between particles, particle size and gradation distribution, etc. [42]. The discrete element method can overcome the limitations of the traditional continuum model theory, and comprehensively simulate the damage process and failure mode of materials from being intact to failure. The damage and failure mechanisms of the material can also be explained from the perspective of micro mechanics [43]. Pervious concrete consisted of granular materials whose different components were not interconnected at the material level. Therefore, DEM was one of the best options for modeling such materials [44,45]. In order to further deepen the understanding of the freeze-thaw damage of permeable concrete, this study used PFC3D discrete element software to establish a freeze-thaw damage model of permeable concrete based on physical experiments, so as to understand the effect of freeze-thaw cycles on meso-parameters such as the bond strength between permeable concrete particles.

Based on the above introduction, this paper deeply explored the freeze-thaw cycle deterioration area of permeable concrete through a combination of macroscopic and microscopic tests. The strength loss, mass loss and relative dynamic elastic modulus change of pervious concrete under freeze-thaw cycles were measured macroscopically, and the strength loss, mass loss and morphology change of hardened cement paste under the same W/C ratio were compared. Microscopically, the morphology evolution of the aggregate–cement interface was explored by a scanning electron microscopy (SEM) test, and the pore structure change of the hardened cement slurry was obtained by a mercury intrusion porosimetry (MIP) test. Therefore, the damage degradation a model undergoing freeze-thaw cycles was proposed. On the basis of the test, a discrete element model of pervious concrete’s freeze-thaw damage was established, which effectively indicated the relationship between the number of freeze-thaw cycles and the bond strength between particles. The research results can enrich the theoretical system of damage mechanisms of pervious concrete under freeze-thaw cycles, and provide theoretical support for pervious concrete construction under freeze-thaw scenarios.

## 2. Materials and Methods

### 2.1. Materials

All mixtures were made of ordinary Portland cement with a strength grade of 42.5. Coarse aggregate was limestone with a particle size of 2.5~10.0 mm, and a particle size of 2.5~5.0 mm accounted for 25% of the limestone. In order to make the pervious concrete in this test similar to the actual pervious pavement material, an SR-5 commercial additive produced by Nanjing Jiuherun Engineering Technology Company was used as the admixture. The recommended additive amount was 3~6% of the cement quality; the additive amount in this test was 4% of the cement quality. According to the supplier′s product introduction, the reinforcer mechanism of the strengthening agent is to participate in the cement hydration reaction to form a high-molecular polymer structure, which can significantly improve the compressive strength and bonding strength of the hydration production. The chemical composition of the additive is shown in Table 1.

According to the Chinese standard CJJ/T 135-2009 [46], the filling theory and volume method were used to calculate the mixture ratio, and the target pore was set as 12%. Previous research results showed that the compressive strength of pervious concrete increased when the water–cement ratio was 0.25–0.31 and decreased when the water–cement ratio was larger than 0.34. When the water–cement ratio was lower than 0.31, the mixture was too dry to affect the molding. When the water–cement ratio was 0.34, it was easy to segregate the paste and affect the water permeability rate. Therefore, a water–cement ratio of 0.31 was used in this test. The amount of each material of pervious concrete was calculated, as shown in Table 2.

### 2.2. Preparation and Maintenance of Samples

(1) Pervious concrete sample

The size of the permeable concrete sample was a 150 mm cube, and the molding adopted a combination of ramming and vibration. The mixture was assembled into the mold three times, and then it was vibrated on a vibrating table for 15 s, and the mold was removed after 24 h. The samples were covered with geotextiles and watered for 28 days.

(2) Hardened cement slurry sample

The ratio of the prepared cement stone material was the same as that of the permeable concrete cementitious material. The sample size was a cylinder with a diameter of 20 mm and a height of 20 mm. The preparation process was that the cementitious material was put into the vessel and fully stirred, and then the slurry was loaded into the mold and then vibrated on a vibrating table for 30 s and placed in the mold for curing. The sample was demolded after 24 h, and the curing was the same as that of the pervious concrete. The above concrete and paste experiments were carried out in a laboratory with a room temperature of 20 °C and humidity of 70%.

### 2.3. Test Methods

#### 2.3.1. Effective Porosity

The pore structure of the pervious concrete was composed of connected pores, semi-connected pores and closed pores, among which connected pores and semi-connected pores belonged to effective pores [47]. Volumetric and gravimetric methods were used to measure the effective porosity of the pervious concrete. This test used the gravimetric method to test the permeability coefficient of the pervious concrete [48]. The gravimetric method was mainly to measure the difference between the drying mass of the sample and the floating weight in water, which can demonstrate the actual buoyancy of the structure after pore saturation. Working on the assumption that the sample does not absorb water, porosity *P* can be obtained by using the difference between actual and theoretical buoyancy. The specimen size was a 150 mm cube and the average value of the three samples was obtained. The calculation formula of porosity *P* was as follows:

(1) $$P=\left[1-\frac{m_{2}-m_{1}}{V}\right]\times 100\%$$

where *P* is the effective porosity (%); *m*_1_ is the floating weight of the sample (g); *m*_2_ is the drying quality of the sample (g); *V* is the sample volume (cm^3^).

#### 2.3.2. Ultrasonic Velocity

Ultrasonic wave velocity was an important parameter in ultrasonic testing of concrete, which was related to the number and complexity of pores in the internal structure of concrete. If there were defects (holes, honeycombs) inside the concrete, the ultrasonic waves will be refracted and diffracted there, resulting in a lower ultrasonic wave velocity. The ultrasonic wave velocity was related to the material components, and the W/C ratio and aggregate content all affected the ultrasonic wave velocity of concrete [49,50]. In a word, the ultrasonic wave velocity of concrete can reflect the performance and internal characteristics of concrete. In order to study the internal structure evolution of pervious concrete under freeze-thaw cycles, ultrasonic tests were carried out on concrete samples with different erosion times, and the changes in ultrasonic wave velocity and density of concrete samples with different erosion ages were tested.

The transmitting and receiving transducers were placed in the center of two opposite surfaces of the pervious concrete. In order to reduce the influence of the gap between the transducer and the concrete surface on the ultrasonic test results, Vaseline was applied as a coupling agent at the contact position between the transducer and the pervious concrete. The ultrasonic detection frequency was set to 28.2 kHz, and the sampling was started after the relevant test parameters were set. When the ultrasonic wave speed and waveform were stable, the sampling was stopped and the data were saved to record the ultrasonic wave speed. The specimen size was a 150 mm cube and the test results were taken as the average value of the three samples.

#### 2.3.3. Compressive Strength

According to the Chinese standard GB/T50081-2002 [51], the 150 mm cubic sample was placed between the upper and lower pressure plates, and the test instrument was started. The loading stress rate was 0.3 MPa/s. The peak load was recorded and the average value of three pervious concrete blocks was obtained.

The calculation formula for the peak compressive strength of pervious concrete is shown in Equation (2):

(2) $$P=\frac{F}{A}$$

where *P* is the compressive strength, MPa; *F* is the peak pressure of specimen failure, kN; *A* is the compressive surface area of the sample, mm^2^.

A strain gauge was applied to the hardened cement paste sample to measure the transverse and longitudinal strain under compression. The strain gauge was connected with the strain gauge, and the upper and lower surfaces of the cement stone sample were coated with butter and placed on the bottom support of the electronic universal testing machine. The test control mode was set as displacement control, and the displacement control speed was set as 0.1 mm/min. The peak load was recorded, and the average strength of three cement blocks was obtained.

#### 2.3.4. Freeze-Thaw Cycle Test

The freeze-thaw cycle test of pervious concrete conforms to the Chinese standard GBT 50082-2009 [52]. There were 5 groups of pervious concrete samples which were 150 mm cubes, and 3 samples in each group. The number of freeze-thaw cycles was set at 100, and damage detection was performed every 25 cycles. The mass and ultrasonic speed of one group were recorded until the final compressive strength test.

The freeze-thaw test block was immersed in the test tank for two days. After it was removed, the test block was wrapped and sealed with plastic wrap until there was no water dripping from the bottom of the test block. Then it was quickly put into the freezer. The freeze-thaw test was started with a freezing temperature of −25 °C and a freezing time for each cycle of 10 h. Then, the test block was put into a water tank (20 °C) to thaw for 4 h.

The specimens were dried after curing for 28 days, and the initial mass of group A was weighed. When group A reached the number of freeze-thaw cycles, the samples were thawed, cleaned with water, and weighed after drying for 24 h, and then their ultrasonic speeds were measured. When the number of freeze-thaw cycles was reached, three samples outside group A were selected for a compressive strength test. After curing for 25 days, the sample was immersed in a 3% sodium chloride solution for three days. After soaking, the sample was removed from the sodium chloride solution, covered with plastic film and left to stand for 10 min to drain the water. The pervious concrete test block was sealed with plastic wrap to prevent evaporation of water during the freeze-thaw cycle. The number of test blocks, method steps, sizes and testing methods of freezing and thawing deterioration in the salt freezing test was consistent with that in the water freezing test. The sample size of the clean cement slurry was a cylindrical sample with a diameter of 20 mm and a height of 20 mm. There were 8 groups of samples with 3 pieces in each group. Three groups were tested for mass loss (25 measurements per freeze-thaw cycle) and compressive strength (50 measurements per freeze-thaw cycle). The other 5 groups were subjected to a mercury injection test (25 measurements per freeze-thaw cycle). The freeze-thaw cycle of the cement slurry was consistent with that of pervious concrete.

#### 2.3.5. Mercury Intrusion Porosimetry (MIP) Test

Mercury injection (MIP) of cement paste specimens was measured by an AutoPore IV 9500 automatic porometer in the pressure range of 0.10 to 61,000.00 Psia, and the contact angle between the mercury and the pore surface was θ = 130°. The sample size of the cement paste prepared in this test was 20 mm in diameter and 20 mm in height, which can be directly used for the mercury injection test. Prior to mercury injection testing, MIP specimens were placed in anhydrous ethanol for at least 24 h to dehydrate and terminate hydration. Then, the sample was put into the oven at 45 °C to dry for 24 h until it reached a constant mass so that free water and alcohol evaporation was achieved.

#### 2.3.6. Scanning Electron Microscope (SEM) Test

The scanning electron microscopy (SEM, XRADI410 Versa, ZEISS, Jena, Germany) was used to observe the microstructure of the interface zone between the aggregate and the hardened paste and the paste zone. After the freeze-thaw test was repeated 50 and 100 times, a thin fragment of 5 mm^2^ was knocked off at the aggregate interface. The samples were soaked with anhydrous ethanol to stop hydration. Before the experiment, the samples were taken out and dried to a constant weight at 60 °C for testing.

#### 2.3.7. Discrete Element Method

**Figure 1:**
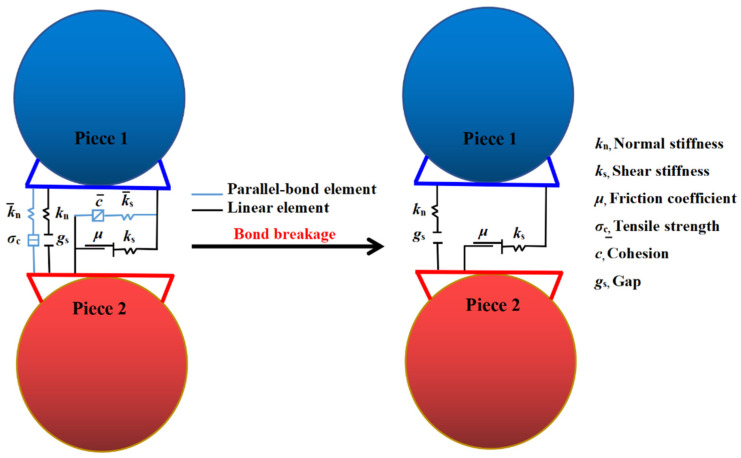
Failure transformation of linear parallel bonding.

The calculation principle of PFC3D discrete element software was mainly based on the force-displacement law and Newton′s second law. It adopted the explicit finite difference method for the cyclic iteration solution and interacted with the internal force and torque. There is bonding strength between particles, and the bond is broken when the force acting on the cohesive bond due to external loads exceeded the strength of the bond itself [40]. In this study, the freeze-thaw damage of pervious concrete was characterized as the damage to the bonds between particles. Therefore, a parallel bond constitutive model, which was similar to the mechanical properties of cementitious materials, was selected to simulate the contact of cement paste between permeable concrete aggregates [53,54]. When the parallel bonding model was bonded, it can resist the torque and behave as linear elasticity when the force did not reach the strength limit. When the force exceeded the strength limit and the load cannot be transmitted after the bond fails, it degenerated into a linear model, as shown in Figure 1 [55].

**Figure 2:**
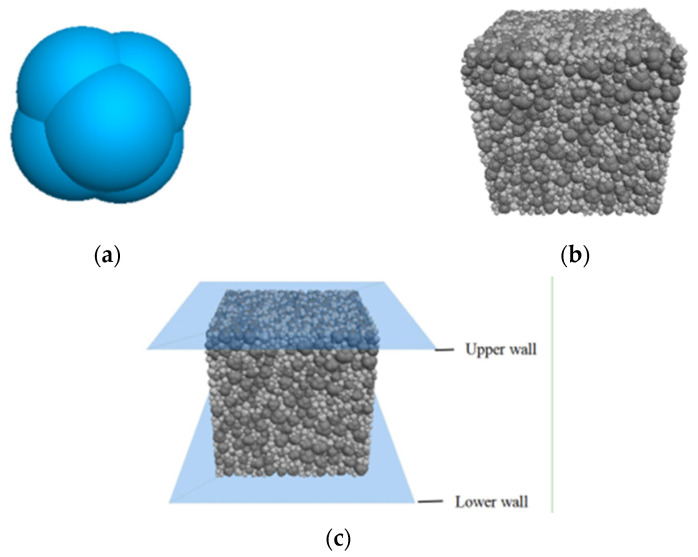
(**a**) Aggregate model (**b**) DEM model of pervious concrete (**c**) Axial loading diagram.

Aggregate is constructed through six discrete particle units embedded with each other, as shown in Figure 2a. The aggregate model is divided into two groups according to particle gradation, with particles of 2.5–5 mm accounting for 25% and particles of 5–10 mm accounting for 75%. The generated model is shown in Figure 2b. The loading rate used in the PFC compressive strength test was different from the loading rate used in a laboratory test. The compressive strength of the traditional loading program was obviously different at various speeds. In the PFC simulation, the time step was 10–8 s and the loading rate was 1.0 m/s. The model should be quasi-static, so the strength value should be unrelated to the loading rate [56]. Since the loading rate did not have a great impact on the model, the loading rate of this test was 0.05 m/s under comprehensive consideration. The axial loading of the DEM model is shown in Figure 2c.

## 3. Results and Discussion

### 3.1. Experimental Results

#### 3.1.1. Macroscopic Performance Deterioration Test Results

##### Surface Morphologic

**Figure 3:**
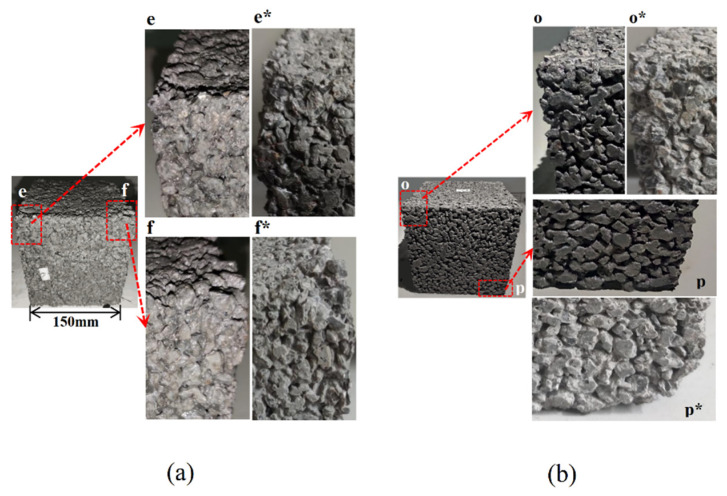
Change of pervious concrete morphology under different freeze-thaw cycles (**a**) aqueous solution; (**b**) salt solution; (e, f, o, p represent F-T 0 times, e*, f*, o*, p* represent F-T 100 times).

Figure 3 shows the morphological changes of pervious concrete in two freeze-thaw environments: water freezing and salt freezing. Cement paste was eroded from the aggregate surface during freeze-thaw cycles. Comparing the changes before and after a freeze-thaw cycle, it was found that the shedding parts of the aggregate were concentrated at the forming surface and corners of the sample, as shown in Figure 3e,f,o,p. It can be seen from Figure 3p that the aggregate paste structure fell off significantly in the lower right corner of the pervious concrete which was salt frozen 100 times. Comparing the damage of water freezing and salt freezing, it was found that salt freezing caused more serious shedding of aggregate paste structure in pervious concrete. These phenomena were the reasons for the quality loss of the pervious concrete.

**Figure 4:**
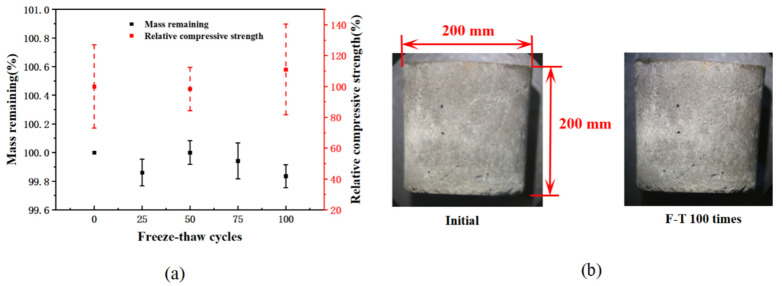
Changes of cement paste under freeze-thaw cycles (**a**) changes in quality and strength; (**b**) changes in morphology.

Figure 4 presents the changes in the cement paste with the same water–cement ratio in the freeze-thaw process. It can be seen from Figure 4b that the freeze-thaw cycle did not cause obvious damage to the cement paste with the same water–cement ratio. During the test process, the macroscopic pore defects on the sample surface were not observed to be significantly aggravated. After 100 freeze-thaw cycles, the mass loss of the cement paste was 0.16%, and the compressive strength was basically unchanged and only slightly increased, as shown in Figure 4a. This indicated that the strength improvement brought by the continued hydration of cement paste was greater than the damage caused by the freeze-thaw cycle. In summary, it can be inferred from the macroscopic properties that the cement paste will not deteriorate significantly after 100 freeze-thaw cycles.

**Figure 5:**
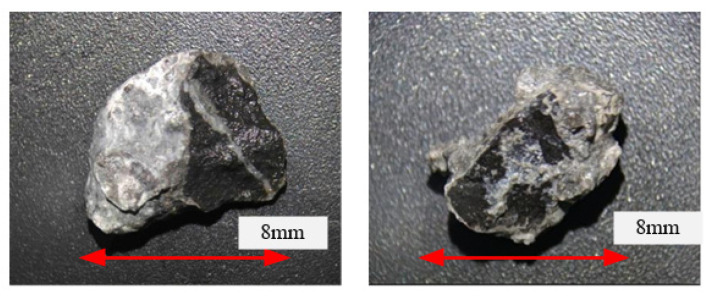
Structure of aggregate paste falling off.

The structure of the dropped aggregate slurry was observed in Figure 5, and it was found that the aggregate surface was clearly visible and smooth. If the freeze-thaw damage was primarily from the cement paste, then the detached aggregate should be encased in the cement paste rather than peeling off directly from the interface. Therefore, it can be considered that the main reason for the quality loss of the pervious concrete was not the deterioration of the cement paste, but the freeze-thaw deterioration of the interface transition zone between the aggregate and hardened paste.

##### Compressive Strength

**Figure 6:**
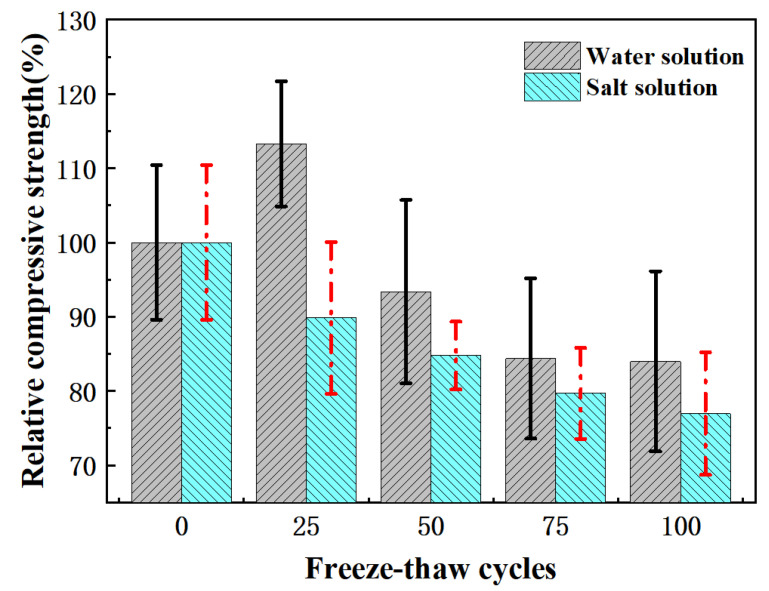
Relative strength change of pervious concrete under freeze-thaw cycles.

The strength changes of the pervious concrete during freeze-thaw under water freezing and salt freezing are shown in Figure 6. The compressive strength of pervious concrete decreased with the increase in freeze-thaw times. After 100 freeze-thaw cycles, the strength of the pervious concrete decreased by 23% in the salt-frozen environment and 16% in the water-frozen environment, indicating that salt freezing did greater damage to the pervious concrete. According to the salt freezing failure mechanism of ordinary concrete, after the concrete was attached with deicing salt, the increases in hygroscopicity led to a greater degree of saturation [57,58]. Moreover, a NaCl solution with a concentration of 2–6% (3% in this experiment) will produce the maximum icing pressure, making the concrete more prone to denudation [59]. Therefore, the damage caused by salt freezing was manifested as a significant attenuation of the mechanical strength. In the water freezing environment, the stress of the pervious concrete increased obviously for the 25th freeze-thaw cycle. The strength of the pervious concrete increased with the increase in hydration degree. Compared with the influence of the freeze-thaw cycle on the strength of concrete, the hydration effect played a leading role.

##### Dynamic Elastic Modulus

**Figure 7:**
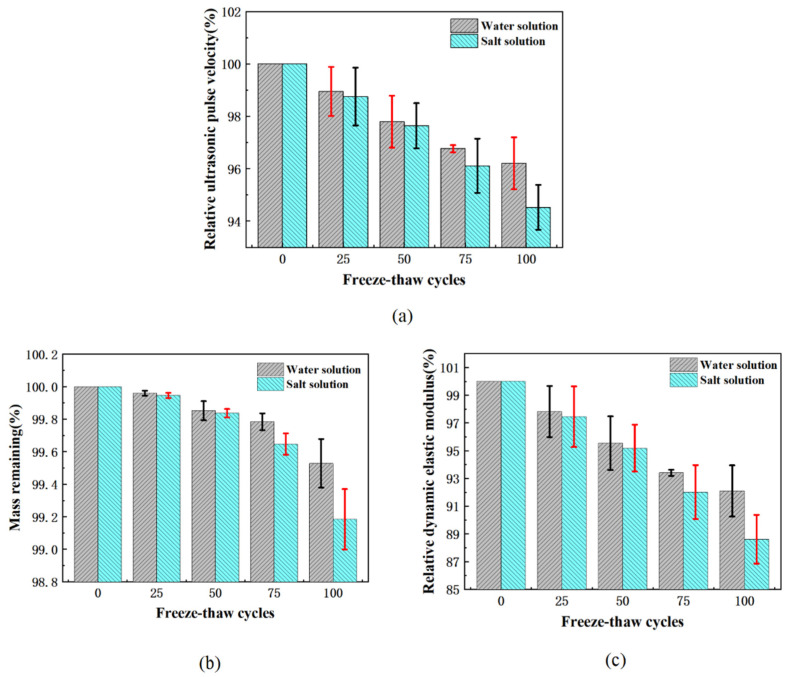
The average index of freeze-thaw durability of permeable concrete (**a**) Relative ultrasonic wave velocity; (**b**) Mass remaining; (**c**) Relative dynamic elastic modulus.

It can be seen from Figure 7a that the ultrasonic velocity of pervious concrete decreased with the increase in freeze-thaw times. Under 100 freeze-thaw cycles, the ultrasonic velocity loss after water freezing was 3.8%, and after salt freezing it was 5.5%. In the freeze-thaw process, the pervious concrete deteriorated continuously, which was characterized by the shedding of cement paste and an increase in micro-cracks and pores. This phenomenon is explained in Figure 3 and the following SEM experiments. As a result, ultrasonic waves needed to undergo more refraction and diffraction in the transmission process, resulting in more complex and changeable transmission paths, which had an increasingly serious impact on ultrasonic waves. Therefore, the increase in the ultrasonic wave velocity attenuation rate will lead to an increase in cracks and pore expansion in the pervious concrete. The attenuation of the wave velocity can provide a reference evaluation for freeze-thaw damage of pervious concrete.

The changes in pervious concrete quality during freeze-thaw cycles in both water and salt freezing environments are shown in Figure 7b. After 100 freeze-thaw cycles, the salt freezing mass loss was 0.82% and the water freezing mass loss was 0.47%. The quality loss was more serious in a salt freezing environment, indicating that deicing salt caused more serious freeze-thaw damage to pervious concrete. However, it should be noted that without freeze-thaw conditions, sodium chloride did not have a significant negative effect on concrete, because the Freidel′s salt produced by the reaction of sodium chloride with cement slurry was not a highly destructive component of concrete [60]. The higher mass loss in a salt-frozen environment was not caused by the chemical erosion of NaCl. The addition of deicing salt gave the pervious concrete higher hygroscopicity, higher saturation, made it reach critical saturation faster, and higher expansion pressure generated by the freezing [61].

Figure 7c shows that after 100 freeze-thaw cycles, the dynamic elastic modulus of pervious concrete in the water-frozen environment and the salt-frozen environment decreased by 7.9% and 11.4%, respectively. The decrease in the dynamic elastic modulus was about half of the strength loss of 100 freeze-thaw cycles. For ordinary concrete, when the strength loss of concrete reached 20%, the loss rates of the dynamic elastic modulus of concrete with water-binder ratios of 0.35, 0.45 and 0.55 were about 3.5%, 7% and 8%, respectively [62]. The effect of freeze-thaw cycles on the dynamic elastic modulus and strength of pervious concrete was similar to that of ordinary concrete. The strength loss was more sensitive than the dynamic elastic modulus under freeze-thaw conditions.

##### Effective Porosity

**Figure 8:**
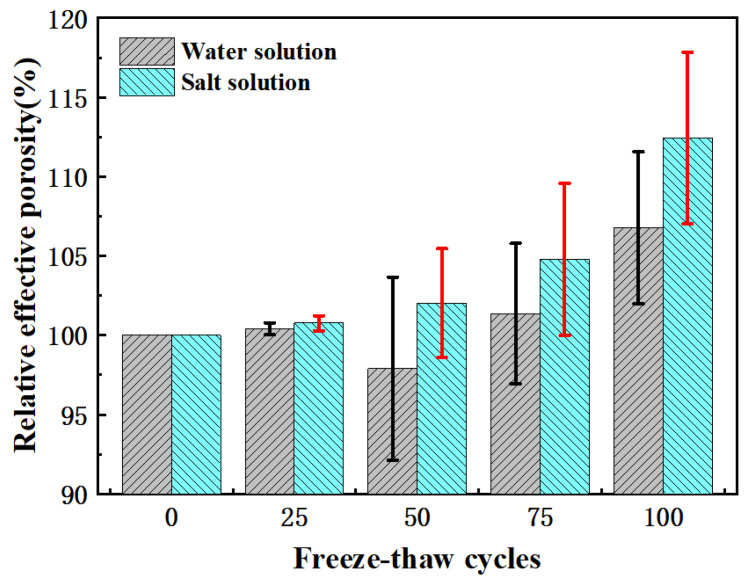
Relative effective porosity change of permeable concrete under freeze-thaw cycles.

Figure 8 shows that the effective porosity of pervious concrete increased with the increase in freeze-thaw times. After 100 freeze-thaw cycles, the effective porosity of salt freezing increased by 12%, and that of water freezing increased by 7%. The main reason was that the aggregate paste structure at the edges and corners of the pervious concrete fell off, and the volume of the aggregate paste structure directly transformed into increased effective pores. In addition, the denudation of cement in the connected pores enlarged the connected pores. As mentioned above, salt freezing was more serious for denudation of pervious concrete, which also explains why the increase in the effective porosity of salt-frozen samples was greater than that of water-frozen samples.

However, it was also found that the fluctuation of the effective pore variation of water freezing in the sample did not seem to come from a measurement error, which may be caused by the coupling agent in the ultrasonic test. The coupling agent Vaseline can fill the surface of pervious concrete and reduce the number of connected pores. The effect of water freezing and salt freezing denudation on effective pores was greater than that of coupling agent filling. Salt freezing was more serious because of mass loss, so this effect was not obvious.

#### 3.1.2. Evaluation of Freeze-Thaw Damage

##### Establishment of a Freeze-Thaw Damage Model

In order to evaluate the deterioration degree of freeze-thaw damage, the quality loss rate, strength loss rate and freeze-thaw damage model based on ultrasonic wave velocity attenuation were used as evaluation indexes, where the mass loss rate *L_m_*, strength loss *L_σ_* and dynamic elastic modulus loss *L_E_* are calculated according to Equations (3)–(5) below:

(3) $$L_{m}=(1-\frac{m_{d}}{m_{0}})\times 100\%$$

(4) $$L_{\sigma }=(1-\frac{\sigma_{c,d}}{\sigma_{c,0}})\times 100\%$$

(5) $$L_{E}=(1-\frac{E_{d}}{E_{0}})\times 100\%$$

where *m*_0_, *σ*_c,0_ and *E*_0_ are the initial mass, compressive strength, and dynamic elastic modulus, respectively, and *m_d_*, *σ_c,d_* and *E_d_* are the mass, compressive strength, and dynamic elastic modulus after reaching the specified number of freeze-thaw cycles, respectively.

Freezing-thawing cycles can cause irreversible damage to pervious concrete, such as cracks, aggregate spalling and fractures, which makes it difficult to guarantee the mechanical properties and service functions of pervious concrete. In damage mechanics, it was generally considered that material damage was irreversible and was a process of energy dissipation. It was believed that the damage to materials was caused by the microscopic defects of materials, resulting in the reduction of the effective bearing area (A) of materials. This microscopic defect process was difficult to measure and needed to be linked to specific measurable macro variables. The continuum (*Ψ*, the ratio of the effective load area before and after the damage) was used to describe the damage state of the material, and the damage variable D (1 − *Ψ*) corresponding to the continuum was introduced. The principle of strain equivalence was proposed, and the damage degree can be expressed by the change of elastic modulus:

(6) $$D=1-\frac{\bar{E}}{E}=1-\frac{\bar{A}}{A}$$

where *E* is the elastic modulus of the material before damage, Gpa; $\bar{E}$ is the elastic modulus of the material after damage, Gpa; $\bar{A}$ is the effective bearing area after material damage.

**Figure 9:**
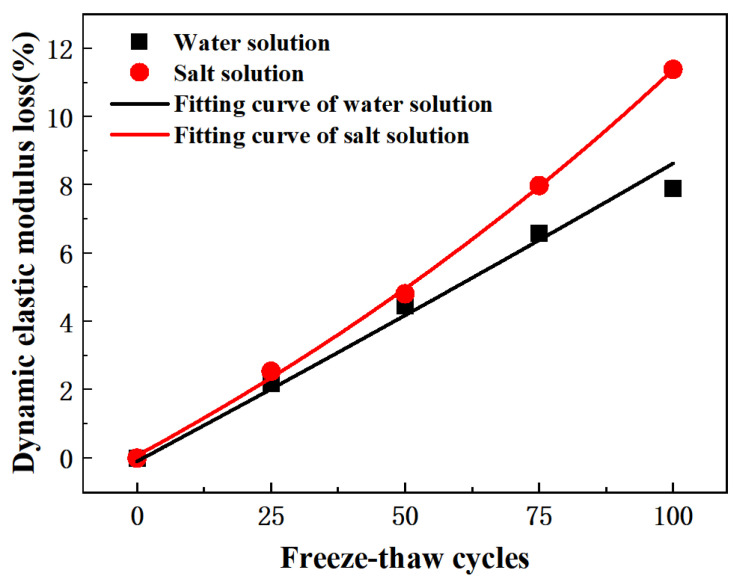
Fitting curve of dynamic elastic modulus loss of permeable concrete under freeze-thaw cycles.

Figure 9 is calculated according to Equation (5). The freeze-thaw damage model of the pervious concrete was constructed using the first-order decay exponential function (ExpDec1), that is, *D_E_* = (*ae*^(−*N*/*b*)^ + *c*)/100, where *D_E_* freeze-thaw damage characterization, *N* was the number of freeze-thaw cycles, *a*, *b* and *c* were undetermined parameters, and the model was as follows:

(7) $$D_{E,w}=(104.83373e^{\left(N/1250.56021\right)}-104.92892)/100,\;R^{2}=0.98337$$

(8) $$D_{E,s}=(15.70047e^{\left(N/184.39276\right)}-15.62693)/100,\;R^{2}=0.99835$$

The degree of damage can be expressed by elastic modulus. Therefore, the freeze-thaw damage model of the water-frozen pervious concrete is represented by Equation (7), and the freeze-thaw damage model of the salt-frozen pervious concrete is represented by Equation (8).

##### Prediction and Evaluation of Freeze-Thaw Durability

Based on the data of 100 freeze-thaw tests, a prediction model of the freeze-thaw quality loss of pervious concrete was established by using the first-order decay exponential function (ExpDec1), namely *D_m_* = (*ae*^(−*N*/*b*)^ + *c*)/100, where *D_m_* is the quality damage characterization, *N* is the number of freeze-thaw cycles, *a*, *b* and *c* are undetermined parameters, and the model is as follows:

(9) $$D_{m,w}=(0.0531e^{\left(N/43.13277\right)}-0.04347)/100,\;R^{2}=0.97391$$

(10) $$D_{m,s}=(0.04114e^{\left(N/33.0477\right)}-0.03528)/100,\;R^{2}=0.99877$$

**Figure 10:**
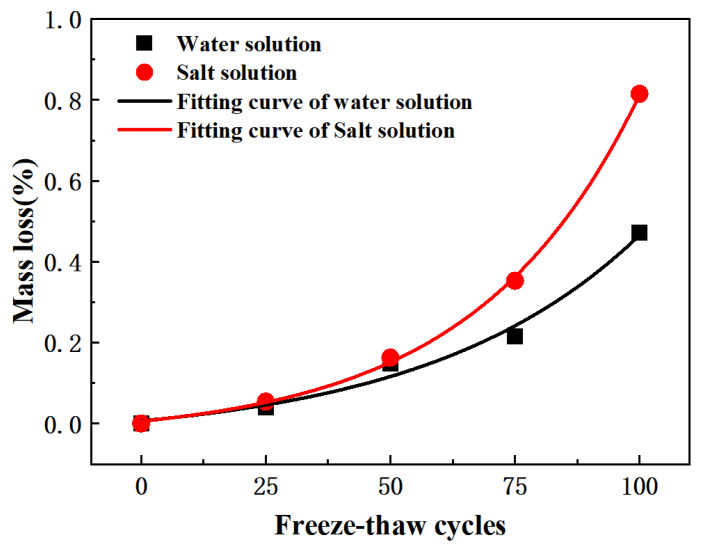
Fitting curve of mass loss of permeable concrete under freeze-thaw cycles.

Equation (9) is the quality loss prediction model of the water-frozen pervious concrete, and Equation (10) is the quality loss prediction model of the salt-frozen pervious concrete. The fitting curve of mass loss of permeable concrete under freeze-thaw cycles is shown in Figure 10.

Due to the dispersion of the 25 freeze-thaw data in the water freezing test, the strength loss model only considered the salt freezing condition. The strength loss model of the pervious concrete is constructed by using the quadratic function (*D_σ_* = *aN*^2^ + *kN*), where *D_σ_* is the strength damage characterization and *a* and *k* are undetermined coefficients. The model is as follows:

(11) $$D_{\sigma ,s}=(0.0001N^{2}+0.25N)/100,\;R^{2}=0.92827$$

**Figure 11:**
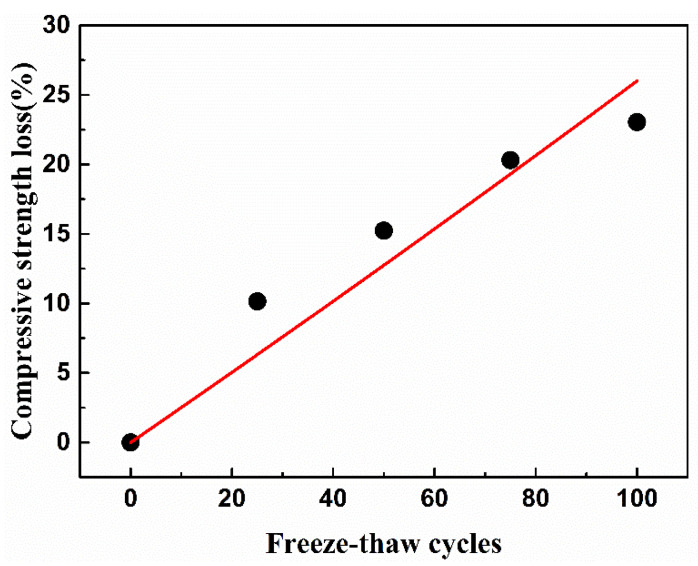
Fitting curve of strength loss of permeable concrete under freeze-thaw cycles.

Equation (11) was the strength loss prediction model of the salt-frozen pervious concrete. The fitting curve of strength loss of the permeable concrete undergoing freeze-thaw cycles is shown in Figure 11. It should be noted that although the regression equation with a higher goodness of fit can be obtained by using other curves here, its deterioration law was not consistent with the reality. The freeze-thaw strength loss of the pervious concrete did not slow down with the increase in freeze-thaw times. In addition, the strength test was different from the nondestructive testing index, which only needed to consider the error caused by testing by using the same group of samples, and it also had discreteness between different samples. Considering the above situation comprehensively, the monotone increasing quadratic function was selected in combination with the error bar range in Figure 6.

### 3.2. Microscopic Property Test Results

#### 3.2.1. Mercury Intrusion Porosimetry (MIP) Results

**Figure 12:**
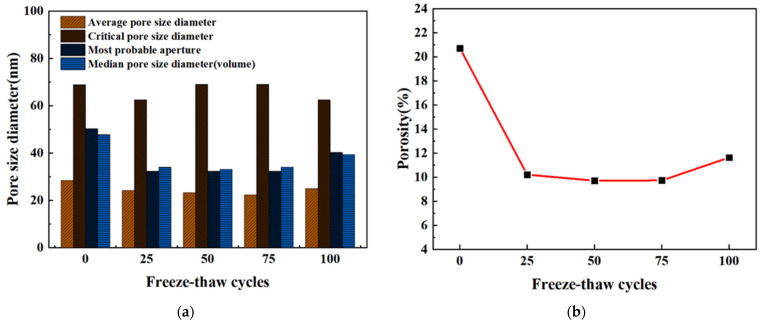
Variation of pore structure characteristic parameters of cement paste with different freeze-thaw cycles. (**a**) Changes of pore size parameter; (**b**) Changes of porosity change.

The variations of various parameters of the cement pore structure with different freeze-thaw cycles are shown in Figure 12. The 100 freeze-thaw cycles had little effect on the four pore size parameters of cement paste at a water–cement ratio of 0.31, which was consistent with the macroscopic properties of freeze-thaw cycles mentioned above. When freeze-thawed 25 times, the porosity of the cement paste decreased by 10% compared with the initial state, which may be due to the formation of hydration products such as CSH and CH in the cement paste, which can effectively fill the pores, therefore making the internal structure of the paste more compact. The porosity remained stable after 25 freeze-thaw cycles, and increased slightly after 100 freeze-thaw cycles.

**Figure 13:**
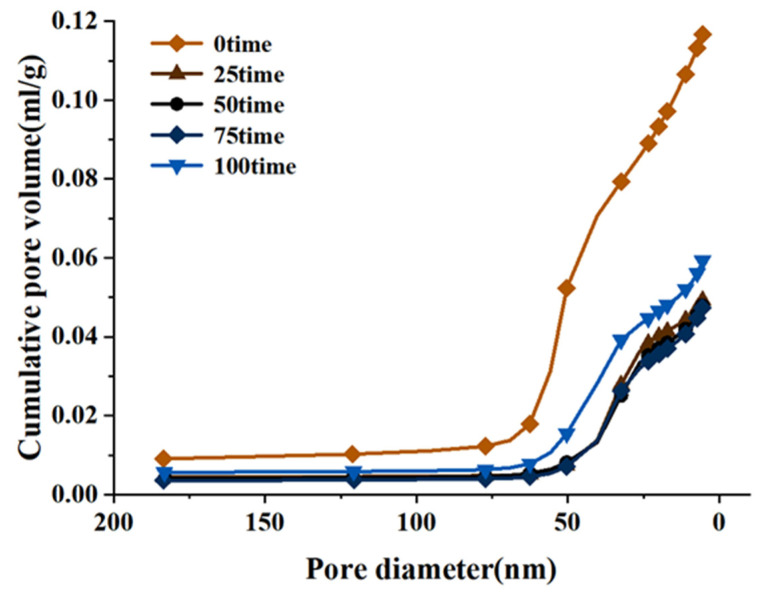
Relationship between pore size distribution and cumulative mercury content of cement paste under different freeze-thaw cycles.

The paste accumulative quantity of different freeze-thaw cycles was shown in Figure 13. With the decrease in pore size, the volume of mercury in the pore size of the cement paste increased in a curve form. When the pore size was in the range of approximately 69–106 nm, there was almost no mercury in the pore size, but the mercury in the pore size below 69 nm increased sharply, indicating that the pore size of the cement paste at a water–cement ratio of 0.31 was mainly concentrated in the range of 5–69 nm.

However, it was also noted that the initial curve also increased greatly at 69 nm, but the final cumulative volume of mercury was about twice that after a freeze-thaw cycle. The paste with a water–cement ratio of 0.31 became denser through the hydration reaction at 5–69 nm during the freeze-thaw cycle. Mehta′s tests showed that pores smaller than 132 nm had no effect on the strength of concrete [63]. This may explain the large porosity of the initial cement paste but the macroscopic strength was not significantly different from that of the freeze-thaw paste.

**Figure 14:**
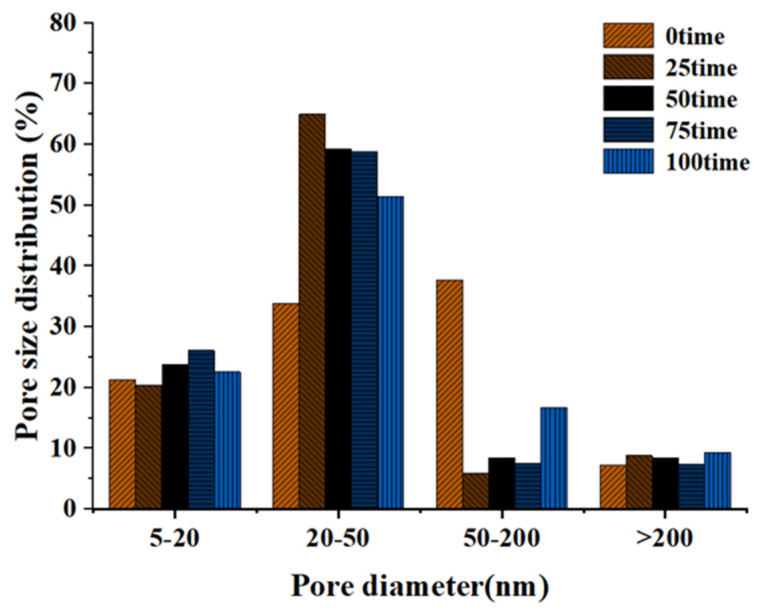
Pore size distribution of cement paste under freeze-thaw cycles.

The pore size distribution of the cement paste with a 0.31 water–cement ratio under different freeze-thaw times was plotted, as shown in Figure 14. The pore size distribution figure clearly showed the change in the small pore size of the freeze-thaw circulation paste. With the increase in freeze-thaw times, pore sizes of 5–20 nm and >200 nm did not change remarkably. Studies had shown that the lower the water–cement ratio, the lower the degree of early hydration [64]. Therefore, in the early freeze-thaw stage, hydration makes the 50–200 nm pores tighter and gradually transformed them into 20–50 nm pores. Later, due to the weakening of hydration, the freeze-thaw cycle played a leading role in the damage caused by the cement paste, and the 20–50 nm pores gradually changed into 50–200 nm pores. The pore size distribution showed that the proportion of the 20–50 nm pores first increased and then decreased, and the proportion of the 50–200 nm pores first decreased and then increased.

Microcapillary pores of 5~100 nm will exhibit the capillary condensation phenomenon, increasing the hygroscopicity of the pores [65]. The higher capillary pressure and osmotic force strengthened the self-shrinkage of the cement paste and accelerated the penetration rate of the cement paste’s surface and atmospheric pressure, thus reducing the impermeability of the cement paste’s surface and atmospheric pressure impermeability. These phenomena enhanced the diffusion of chloride ions and pore water in the cement paste. Although 100 freeze-thaw cycles had no obvious influence on the macroscopic properties and micropore structure of cement paste with a 0.31 water–cement ratio. However, the increase in the transport of the freeze-thaw medium may have caused the freeze-thaw damage to the pervious concrete′s interfacial transition zone (ITZ) of the paste and the aggregate.

#### 3.2.2. Scanning Electron Microscope (SEM) Results

**Figure 15:**
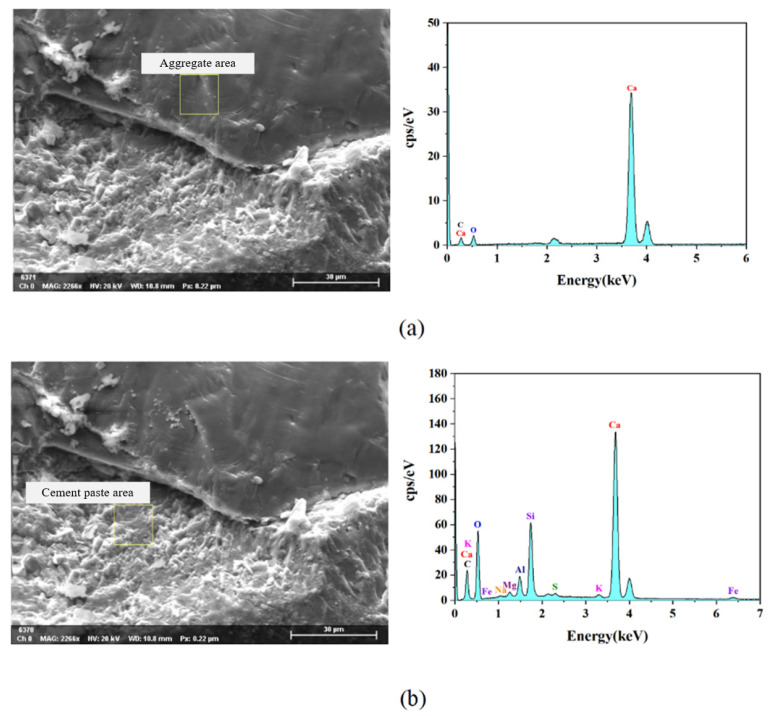
EDS spectrum of ITZ (**a**) aggregate area; (**b**) hardened cement paste area.

The components of the aggregate paste structure can be determined by EDS analysis. Figure 15 shows the EDS spectra of the aggregate and cement paste area, respectively. The various element content of aggregate and hardened cement paste is described in Table 3. The results showed that the aggregate elements in the aggregate paste structure were Ca, O and C, and the content of Ca was the highest because the aggregate was mainly composed of limestone, and the limestone was composed mainly of calcium carbonate (CaCO₃). In addition to the elements contained in the aggregate, there were other elements such as Al and Fe in the cement paste, which proved that the observation area was actually the interface area between the aggregate and the cement.

**Figure 16:**
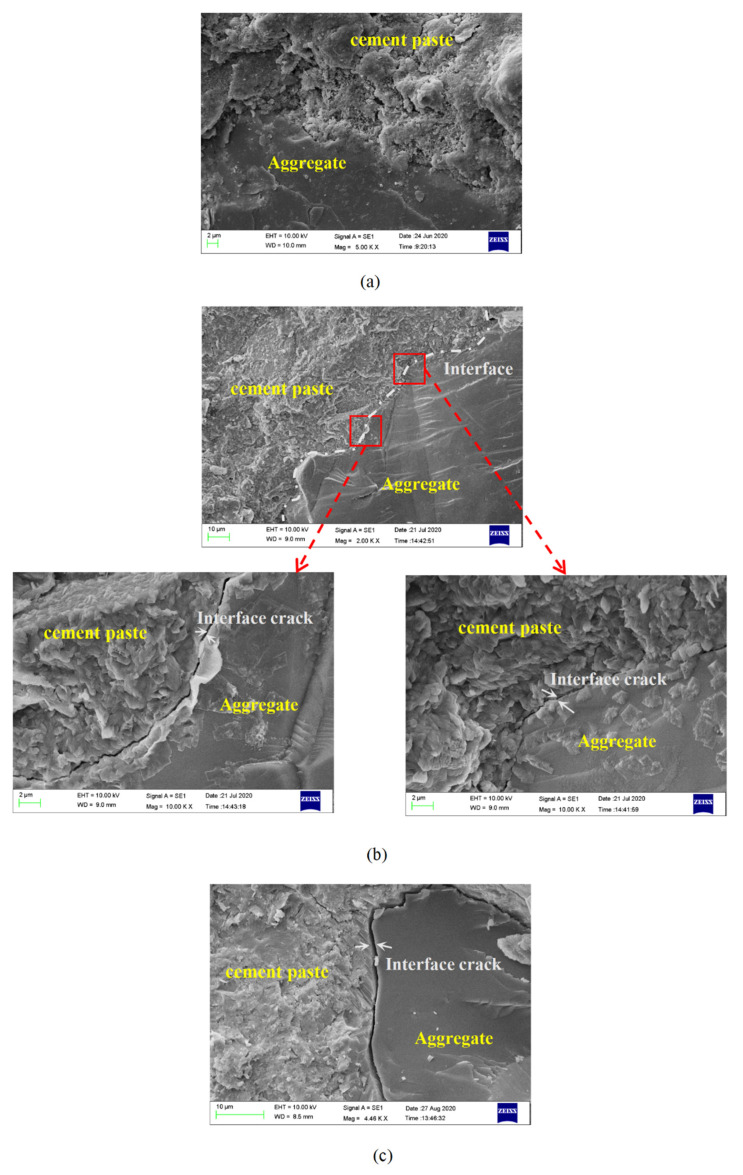
SEM image of aggregate interface with different freeze-thaw cycles (**a**) Initial; (**b**) F-T 50 times; (**c**) F-T100 times.

Figure 16 shows the microscopic morphology of the interface area between the aggregate and the cement paste during a freeze-thaw cycle. According to Cwirzen et al. [66], ordinary concrete with a water–cement ratio of 0.3 had a dense cement paste structure, and electron microscopic observations of these concretes confirmed the existence of an almost undetectable transition zone (less than 5 μm wide), while the interface zone of the sample with a 0.42 water–cement ratio was 40 μm. This experiment showed similar observation results. As the pervious concrete did not contain sand, its transition zone was denser and more difficult to observe than ordinary concrete. There was almost no interface transition zone in the pervious concrete with a water–cement ratio of 0.31.

The interface area of aggregate and cement paste showed great differences under different freeze-thaw cycles. With the increase in freeze-thaw times, the two-phase contact area of aggregate and hardened paste deteriorated obviously. In the unfreeze-thawing of the concrete sample, the interface between aggregate and cement paste was smooth. It can be seen by comparing Figure 16a,b that after 50 freeze-thaw cycles, obvious cracks appeared at the interface between the two phases, and the aggregate and paste structure separated. The elastic modulus and temperature sensitivity of the two-phase materials between the aggregate and the cement paste were different, which produced different shrinkages and expansions during freeze-thaw cycles. The uncoordinated material deformation caused high interfacial stress in the interface area, which caused the obvious expansion of micropores or cracks in the interface area, and finally led to the interface cracking and delaminating. The long and narrow interface cracks speed up the entry of freeze-thaw media and further accelerated the deterioration of the interface area. It can be seen from Figure 16c that the interface cracks with 100 freeze-thaw cycles expanded further than those with 50 freeze-thaw cycles. The presence of an interfacial zone promoted the diffusion of chloride ions, and the diffusion rate was 6–12 times faster than that of the cement paste zone [67]. Therefore, the presence of the interface zone facilitated the entry of harmful ions from the external environment, leading to various harmful chemical reactions, such as chemical erosion and salt-freezing damage of concrete from deicing salts.

### 3.3. Establishment of Freeze-Thaw Damage in DEM

The failure process of pervious concrete under freeze-thaw cycles is essentially the process of internal deterioration of the material. Microdefects in the pervious concrete can be regarded as a damage field continuously distributed within the material. In the freeze-thaw cycle, freeze-thaw damage is constantly generated and expanded, which reduces the strength, stiffness, service function and residual life of the pervious concrete material [68]. Therefore, the effect of the freeze-thaw cycle on the microstructure parameters of the bond affected the microstructure parameters of the whole model.

The DEM simulated the macroscopic mechanical response of concrete by means of the law of bond contact at the particle scale, so the macroscopic material parameters obtained through experiments cannot be directly applied to the numerical model. The DEM simulated the macroscopic mechanical response of concrete by means of regular bond contact at the particle scale, so the macroscopic material parameters obtained through experiments cannot be directly applied to the numerical model [69].

The compressive elastic modulus and peak compressive strength of the parallel bond model materials were affected by the effective modulus of the parallel bond, normal bond strength and tangential bond strength [70,71]. The formula for compression modulus *E_t_* is as follows:

(12) $$E_{t}=\frac{\sigma_{c}}{\varepsilon_{c}}$$

where *E_t_*is the compression–elastic modulus, Gpa; *σ_c_* is the peak stress; *ε_c_* is the peak stress corresponding strain.

**Figure 17:**
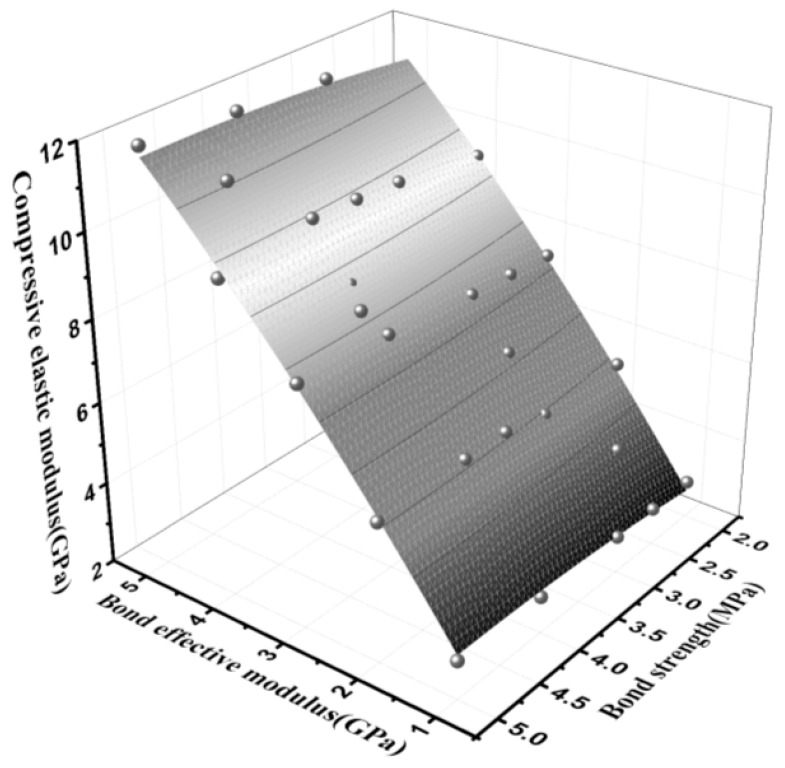
Relationship between bond effective modulus, bond strength and compressive elastic modulus.

**Figure 18:**
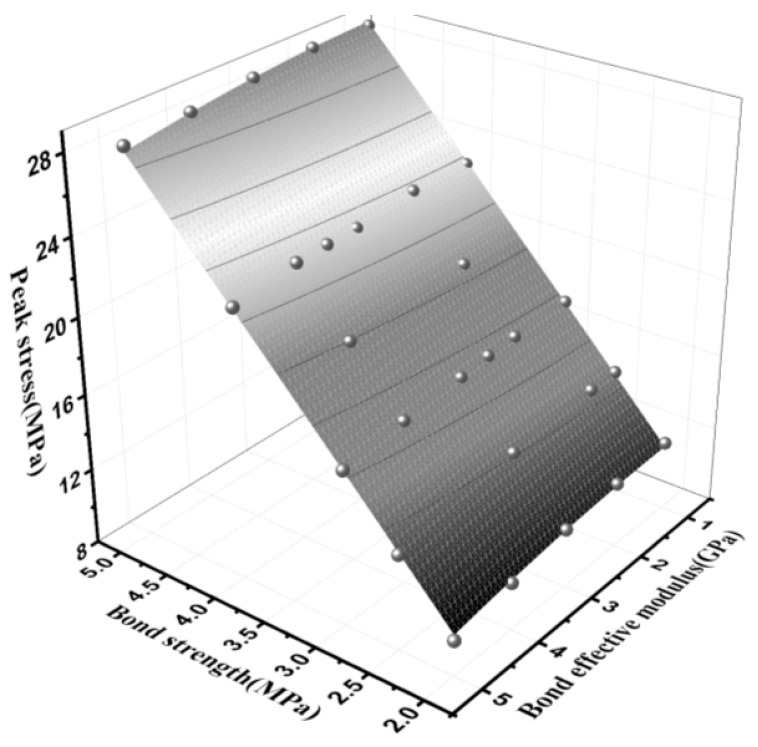
Relationship between bond effective modulus, bond strength and peak stress.

In this model, the bond strength ratio of normal shear was set as 1:1, and the two were collectively referred to as bond strength. Both effective modulus and compressive strength tests were performed to obtain the relation between the effective modulus and compressive modulus, as shown in Figure 17. Figure 18 presents the relationship between the bond effective modulus, bond strength and peak compressive strength and the fitting equation was established, as shown in Equation (13). The bond effective modulus and bond strength were the main influencing factors of the compressive modulus and peak stress, respectively. At the same time, the change of the bond effective modulus had a slight influence on the peak stress, and the change of the bond strength also had the same effect on the compressive elastic modulus. However, for a more accurate simulation, the interaction of the two should be considered using macro parameters.

(13) $$\left\{ \begin{array}{l} E_{t}=0.52165\bar{c}-0.055{\bar{c}}^{2}+2.73867\bar{E^{*}}-0.09931{\bar{E^{*}}}^{2}-0.7854,\;R^{2}=0.99761\\ \sigma_{c}=6.08544\bar{c}-0.03441{\bar{c}}^{2}+0.05666\bar{E^{*}}-0.06233{\bar{E^{*}}}^{2}-0.5919,R^{2}=0.99949 \end{array}\right.$$

where *E_t_* is the compression–elastic modulus, Gpa; $\bar{E}$^*^ is the effective modulus of parallel bonding, Gpa; *σ_c_* is the peak stress (compressive strength), MPa; $\bar{c}$ is bond strength, MPa.

It can be seen from Equation (13) that in order to establish the relationship between the number of freeze-thaw cycles and the meso-parameters of the model, the relationship between the compressive elastic modulus *E*_t_, the peak stress *σ*_c_ and the number of freeze-thaw cycles should be established first. Since only the initial peak strain was measured in this study, so the relationship between the relative peak strain of concrete and the number of freeze-thaw cycles and the cube′s compressive strength (peak stress) is shown as follows [72]:

(14) $$\frac{\varepsilon_{c,D}}{\varepsilon_{c,0}}=(-7.57\sigma_{c,0}+408.85)N\times 10^{-4}+1$$

where *ε_c,D_* is the compressive peak strain of concrete after freeze-thaw; *ε_c_*_,0_ is the compressive peak strain of unfreeze-thawed concrete; *σ_c_*_,0_ is the compressive strength of unfreeze-thawed concrete; *N* is the number of freeze-thaw cycles.

The relationship between the peak stress *σ_c_* and the number of freeze-thaw cycles can be obtained according to the previous strength loss model Equation (11), the following Equation (15) can be obtained:

(15) $$(1-\frac{\sigma_{c,s,d}}{\sigma_{c,0}})\times 100\%=(0.0001N^{2}+0.25N)/100$$

According to Equations (14) and (15) and the initial compressive strength of freeze-thaw 23.13 MPa, the measured peak strain was 2.78 × 10^−3^. The compressive stress and strain of the concrete undergoing freeze-thaw cycles in a salt freezing environment are shown in Table 4.

Equations (12)–(15) were combined to obtain the relationship between the number of freeze-thaw cycles N and the microscopic parameters of the discrete element model, as shown below:

(16) $$\left\{ \begin{array}{l} \frac{23.13-2.313\times 10^{-5}N^{2}-0.057825}{5406.77395N\times 10^{-4}+23.13}=0.52165\bar{c}-0.055{\bar{c}}^{2}+2.73867\bar{E^{*}}-0.09931{\bar{E^{*}}}^{2}-0.7854\\ 23.13-2.313\times 10^{-5}N^{2}-0.057825N=6.08544\bar{c}-0.03441{\bar{c}}^{2}+0.05666\bar{E^{*}}-0.06233{\bar{E^{*}}}^{2} \end{array}\right.$$

**Figure 19:**
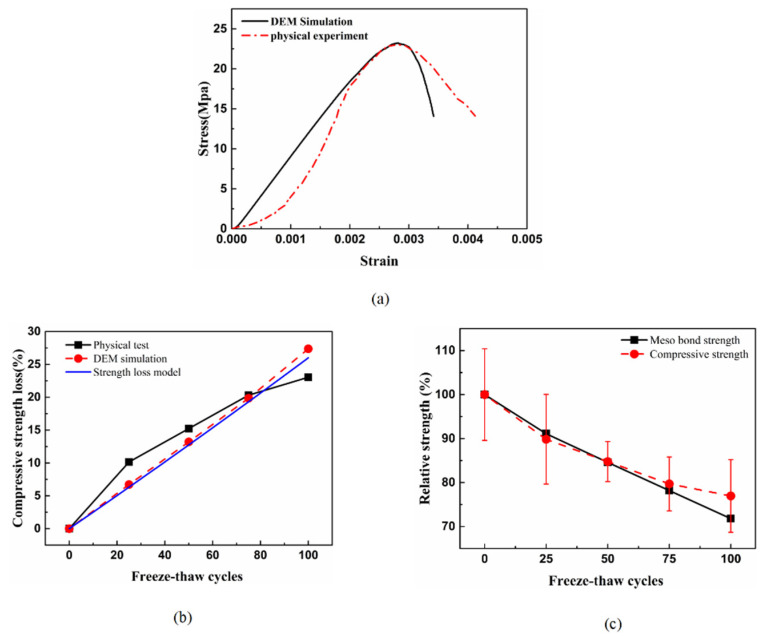
(**a**) Comparison of initial stress and strain curves of pervious concrete by discrete element simulation; (**b**) Comparison of strength loss DEM simulation value and test value; (**c**) Comparison of relative compressive strength and relative meso bond strength.

We substitute the freeze-thaw times *N* of 0, 25, 50, 75 and 100 into Equation (16) to obtain the model′s microscopic parameters under different freeze-thaw times, as shown in Table 5. The mesoscopic parameters of different freeze-thaw times were input into the established discrete element model. After PFC software calculation, the initial stress–strain curve simulation curve and the compressive strength values of different freeze-thaw cycles are obtained, as shown in Figure 19a,b.

It can be seen from Figure 19a that there was a certain deviation between the physical test results and the DEM simulation results. The physical compressive strength test of the permeable concrete initially had a compaction stage, and then it was linearly elastic. The DEM simulation directly entered the linear elastic stage, and the DEM simulation of the descending section of the post-peak curve was more advanced. Such deviations may arise from the simplifications assumed in the constitutive model of this model, or due to imperfections in the contact between the specimen and the loading plate in the experimental tests [73]. However, this error did not affect the accuracy of compressive elastic modulus and peak stress. It can be seen from Figure 19b that the DEM simulation value of the compressive strength of pervious concrete with different freeze-thaw times had some deviation from the experimental value, and the existence of such a deviation was reasonable. This was because the discrete element freeze-thaw damage model was established based on the regression equation of test strength loss, as shown in Equation (11), and the deviation between the regression equation and the test value resulted in the deviation of the discrete element fitting value. It can be seen that DEM simulation intensity loss was close to the intensity loss model. The previous section explained why model curves that fit better with experimental values were not selected. The relationship between the mesoscopic bond strength and macro strength of the pervious concrete is shown in Figure 19c. The variation of the mesoscopic bond strength of the model undergoing freeze-thaw cycles was highly consistent with the variation of the macroscopic bond strength in the physical test, indicating that the strength deterioration of the pervious concrete came from the reduction of bond strength between the aggregate particles. Although the DEM model had a good simulation of the strength changes of pervious concrete under freeze-thaw cycles, the model was simplified in the following aspects. In the simulation, the ratio of normal and tangential bond strengths was fixed, and the failure modes of specimens were not compared and calibrated. The coarse aggregate was a rigid body without fracture and there was a single aggregate shape. This also led to the limited applicability of the model in the study of other problems, such as the study of failure morphology changes of pervious concrete with different cycles.

## 4. Conclusions

In this study, through freeze-thaw cycle tests (water freezing and salt freezing) on pervious concrete and paste with the same W/C ratio, the mass loss, dynamic elastic modulus and compressive strength attenuation laws were investigated, and the freeze-thaw damage prediction model of pervious concrete was established. The relationship between the freeze-thaw damage of a hardened cement paste and the freeze-thaw damage of pervious concrete was studied. At the micro-scale, the evolution of porosity, characteristic pore size and pore size distribution of frozen and thawed paste (at the same W/C ratio as pervious concrete) was studied by the mercury intrusion porosimetry (MIP) method. The damage process and mechanism of the freeze-thaw cycle (water freezing and salt freezing) on the microstructure of pervious concrete were analyzed by scanning electron microscopy (SEM) and energy dispersive spectrometry (EDS). The relationship between the mesoscopic parameters and the macroscopic properties of the pervious concrete specimens was analyzed by the discrete element method (DEM) in combination with the experimental mixed-ratio parameters. The conclusions of this paper were as follows:

After 100 cycles of freezing and thawing, the mass loss of the pervious concrete was 0.47% and 0.82%, the strength loss was 16% and 23% and the dynamic elastic modulus loss was 7.9% and 11.4%, respectively. The water–cement ratio did not change the strength significantly. Considering the interfacial crack propagation observed by electron microscopy, it can be concluded that the freeze-thaw deterioration of the pervious concrete mainly came from the interfacial zone.

The pore size of the cement paste with a water–cement ratio of 0.31 was concentrated at 5–69 nm, and the change of pore size distribution below 200 nm did not affect the macro-strength of the cement paste. There is almost no aggregate cement interface zone in pervious concrete at a water–cement ratio of 0.31.

The DEM model can better simulate strength changes of pervious concrete undergoing freeze-thaw cycles, and its macroscopic strength changes were consistent with the microscopic bond strength changes between particles in the DEM model, indicating that the strength deterioration of pervious concrete was caused by the reduction of the bond strength between the aggregate particles. However, due to the limitation of the assumptions of the model, this model was only applicable to the study of strength loss, and it had limitations in the study of other freeze-thaw deterioration indexes or the change of failure forms of pervious concrete. Therefore, the model can be further improved in the future, and more mesoscopic parameter changes need to be considered and calibrated with physical tests in terms of failure modes.

## Figures and Tables

**Table 1:** Performance parameters of Jiuherun brand additive agent.

Solid Content/%	Moisture Content/%	Fineness/%	Total Alkalinity/%	NaSO_4_/%	CaO/%	SiO_2_/%	Bulk Density/g/cm^3^
96	3.17	16.87	6.46	4.83	0.92	64.6	0.678

**Table 2:** Amount of each material per cubic meter of permeable concrete.

Cement kg/m^3^	Aggregate kg/m^3^	Water kg/m^3^	Reinforcer kg/m^3^
417.28	1600	129.35	16.69

**Table 3:** Contents of each element in energy spectrum.

Energy Spectrum Analysis Region	Element (wt%)						
	O	Ca	C	Si	Al	Fe	Other
Aggregate	25.64	68.13	6.23	-	-	-	-
Cement paste	45.83	28.73	15.42	5.68	1.91	0.75	1.68

**Table 4:** Compressive stress and strain values of concrete under freeze-thaw cycles (salt freezing).

Number of Freeze-thaw Cycles (N)	0	25	50	75	100
Peak stress (MPa)	23.13	21.67	20.18	18.66	17.12
Peak strain (×10^−3^)	2.78	4.38	6.03	7.65	9.28

**Table 5:** Changes of meso parameters of model under freeze-thaw cycles.

Number of Freeze-thaw Cycles	Bond Effective Modulus (Gpa)	Cohesion (MPa)	Bond Normal-to-Shear Stiffness Ratio	Friction Angle (°)	Fric
0	3.22	4.120	1.5	40	0.5
25	1.776	3.754	1.5	40	0.5
50	1.136	3.485	1.5	40	0.5
75	0.795	3.221	1.5	40	0.5
100	0.585	2.958	1.5	40	0.5

## Data Availability

Data Sharing is not applicable.
